# Chest drain through utility vs separate incision in single-port video-assisted thoracoscopic lobectomy: a randomized controlled trial

**DOI:** 10.1097/MS9.0000000000003233

**Published:** 2025-04-10

**Authors:** Fahmi H. Kakamad, Bnar J. Hama Amin, Abdulwahid M. Salih, Shvan H. Mohammed, Hiwa O. Baba, Gasha S. Ahmed, Soran H. Tahir, Rawezh Q. Salih, Berun A. Abdalla, Dahat H. Hussein, Suhaib H. Kakamad, Hussein M. Hamasalih, Mohammed Subhan Mohammed

**Affiliations:** a College of Medicine, University of Sulaimani, Sulaymaniyah, Iraq; b Kscien Organization for Scientific Research (Middle East Office), Sulaymaniyah, Iraq; c Scientific Affairs Department, Smart Health Tower, Sulaymaniyah, Iraq; d Department of Medical Laboratory Science, College of Health Sciences, University of Human Development, Sulaymaniyah, Iraq; e Research Center, University of Halabja, Halabja, Iraq

**Keywords:** chest tube, lobectomy, single utility port, uniport, video-assisted thoracoscopic surgery

## Abstract

**Background:**

This study explores whether using a separate incision for pleural drainage will yield superior results compared to inserting a chest drain through the utility incision in patients with operable lung cancer undergoing single utility port video-assisted thoracoscopic lobectomy.

**Research question:**

Which one is the best?! Inserting the drain through the utility incision or separate incision in a single utility port video-assisted thoracoscopic lobectomy for lung cancer.

**Materials and methods:**

This was a randomized, open-label, superiority trial including patients with bronchogenic carcinoma who underwent a single utility port video-assisted thoracoscopic lobectomy over a 32-month period. The primary outcome was wound infections and postoperative pain, while the secondary outcomes were the duration of hospitalization and return to work.

**Results:**

The study included 89 patients, and 80 patients completed the trial. The mean age was 65.35 ± 9.47 years, 64 (80%) patients were male, and 69 (86.25%) patients had a 0 ECOG score. Eleven patients (13.75%) had a score of 1. The majority of the patients (35%) were in stage IIIA. There was a small but statistically significant difference in return to work when comparing group A with group B (34 ± 6 vs 31 ± 4 days, *P* = 0.038). More important were the differences in patients with wound discharge (29 vs 4; *P* < 0.001) and especially the need for intravenous antibiotic treatment (12 vs 1; *P* < 0.002). There was only a minimal trend for a decrease in the duration of hospitalization. There was no significant difference in postoperative pain scores.

**Conclusions:**

Inserting the chest drain through a separate incision has superior results compared to inserting it through the utility incision in patients with operable lung cancer undergoing single-port video-assisted thoracoscopic lobectomy. However, more studies with a larger sample size are necessary to confirm these results.

**Clinical trial registration:**

The research was registered in the Research Registry. The registration number is researchregistry8426. The link is https://www.researchregistry.com/register-now#home/?view_2_search=researchregistry8426&view_2_page=1.

## Introduction

Bronchogenic cancer (BGC) is a widespread disease and one of the leading causes of mortality worldwide^[1,2]^. Since the early 1990s, video-assisted thoracoscopic surgery (VATS) has been practiced for the management of BGC^[^3^]^. Currently, VATS has become the mainstay approach for the resection of early-stage mediastinal and pulmonary cancers because it has been shown to be more advantageous in comparison to conventional thoracotomy due to better cosmetic outcomes, less postoperative pain, and faster recovery^[^4,5^]^. With increased interest in this treatment modality over the last 25 years, VATS techniques have rapidly evolved, moving from conventional multi-port VATS to less invasive single-port (single-incision) VATS^[^6,7^]^.

**HIGHLIGHTS**

- Video-assisted thoracoscopic surgery (VATS) has become the mainstay approach for the resection of early-stage mediastinal and pulmonary cancers.
- Despite the progress that single-port VATS offers, there are still some associated controversies.
- With single-port VATS, the chest tube is usually placed through the same incision as the VATS utility port.
- No one has compared inserting a chest tube through the utility incision vs its insertion through a separate incision.
- This study aims to evaluate whether using a separate incision for pleural drainage will yield superior results compared to inserting the chest tube in the utility incision after single-port VATS lobectomy for bronchogenic cancer.

VATS is constantly being refined and improved, and currently, it is considered a crucial element of enhanced recovery after surgery (ERAS). This concept refers to a recent, widely accepted evidence-based surgery protocol that aims to enhance recovery and decrease postoperative complications and the duration of hospitalization^[^8,9^]^. Despite the progress that single-port VATS offers, there are still some associated controversies, most notably the ideal site for the insertion of a chest tube after the completion of the operation^[^10,11^]^. While with single-port VATS, the chest tube is usually placed through the same incision as the VATS utility port, the best strategy for chest tube management has yet to exist^[^8^]^. Although prospective and retrospective investigations have been conducted on various approaches for chest tube management after VATS, no one has compared inserting a chest tube through the utility incision to its insertion through a separate incision.

The aim of this study was to evaluate whether using a separate incision for pleural drainage would yield superior results compared to inserting the chest tube in the utility incision after a single-port VATS lobectomy for BGC.

## Materials and methods

### Study design and setting

This was a single-center, pilot, open-label, parallel-arm, randomized controlled trial (RCT) designed to examine the superiority of inserting a single chest tube through a separate incision vs inserting the tube through the utility incision of a single-port VATS. All patients had a resectable BGC or suspected BGC and were to undergo a lobectomy. The trial was conducted according to the Declaration of Helsinki. The study proposal was accepted by the scientific and ethical committee. All participants provided informed consent before admission to the trial. Patient recruitment was carried out between 1 October 2020 and 1 June 2023. The reporting of the study adhered to the principles and guidelines of Consolidated Standards of Reporting Trials (CONSORT) 2010^[^12^]^.

### Participants

The trial population comprised eligible patients aged 30–80 years. All had an Eastern Cooperative Oncology Group performance (ECOG) score of 0 or 1 and primary operable non-small cell lung cancer (stages I–IIIA) and were scheduled to undergo single-port VATS lobectomy. The stage of the disease was determined according to the eighth edition of the TNM classification of malignancy^[^13^]^. Participants were excluded from the study perioperatively if they met any one of the following criteria: (1) patients with diabetes mellitus or connective tissue disease; (2) use of immunosuppressive drugs; (3) history of radiotherapy; (4) active fungal or bacterial infection; (5) concomitant malignancies; (6) women who were pregnant; (7) malnourished patients (body mass index of less than 18); (8) resection of more than one lobe; (9) coagulopathy of any form, including use of or needed for anti-coagulants; (10) history of previous chest operation; (11) conversion of VATS to open thoracotomy; (12) prolonged air leak (lasting more than 5 days); (13) intraoperative decision leading to additional resection; and (14) presence of severe pleural adhesions during surgery.

### Randomization and blinding

Once confirmed to be eligible, initial registration was performed by admitting the patient’s electronic file to a specific inbox in Smart Health Tower’s database. The second registration was done after confirming all preoperative conditions for inclusion using an electronic assignment. A member of the research team randomly generated allocation sequence codes (1:1 allocation ratio) by using SAS statistical software and simple random sampling to randomly assign the patients into two groups: group A (inserting the chest tube through the VATS utility incision) or group B (inserting the chest tube through a separate incision). Patients were randomized to one group or the other exactly when they were admitted to the ward preparing for operation. A minimization method was applied to balance the groups according to age, sex, type of resection, and histological type (squamous cell cancer, adenocarcinoma, or others). The third and final registration was done when the patients were discharged home and met all criteria for inclusion in the study. The nature of the intervention did not allow blind randomization; thus, no blinding of investigators or participants to the allocation was performed.

### Procedure

All the enrolled patients were planned to undergo lobectomy through a single-port VATS. The procedure was performed under general anesthesia in a lateral position (right or left) through an utility incision (4–5 cm in the fifth intercostal space, centered in the posterior axillary line). Hilar and mediastinal lymph node dissections were also performed. After completion of the procedure, in group A, a chest tube (size 28 French) was inserted under vision through the same VATS utility incision, while in group B, after completion of the procedure, the chest tube was inserted through a separate incision in the anterior axillary line, seventh intercostal space. The patients were extubated in the operating room and admitted to the surgical ward with a daily dressing of the wounds. A Numerical Rating Scale (NRS) of 0–10 was used to assess postoperative pain (mild: ≤3, moderate: 4–6, severe: ≥7) for the first week after the operation. Assessment for the pain was done twice a day at 9:00 a.m. and 9:00 p.m. The mean of the total score was calculated. Just before discharge, a wound swab was taken from any discharge at the utility wound (if present) and sent for culture and sensitivity testing. When infection was encountered, culture-directed antibiotics were given orally, and intravenous antibiotics were given whenever white blood cell was increased above normal (>11 000). The analgesic requirement was calculated for both groups. The patients received an acetaminophen infusion (1000 mg × 3) and ketorolac (30 mg × 3 IV); if they still complained of severe pain, morphine (50 mg, slow IV) was added. If the patient still complained of pain, pethidine 50 mg was administered subcutaneously. The Data Safety Monitoring Board (DSMB) monitored the safety of the intervention in both groups. DSMB members were the authors of the study because they also formed a significant part of the administration of the center. The DSMB had the plan to meet after 30%, 50%, and 75% of the patients’ randomization and any time when serious complications (like the requirement of reoperation) would happen.

#### Follow-up

Criteria for chest tube removal were: (1) at least 48 hours passed after the discharge from the operating room. (2) Absence of an air leak or pneumothorax. (3) Minimal drainage (less than 200 cc per 24 hours). The orifice of the chest tube site was closed by a single figure of eight stitches using two zero-polypropylene suture materials. The patients were seen 1 week after discharge; later, they were seen 6 weeks after the operation. Phone-call follow-up was continued for 3 months after the operation. They were contacted once a month.

### Outcome measures

The primary outcomes were wound infection rate and postoperative pain, while the secondary outcomes were the duration of hospitalization and return to work (for the retired patients, return to work was assumed whenever the patient started a normal lifestyle as preoperative days). The NRS score was used for measuring postoperative pain. Patients with mild pain (≤3) were managed by oral analgesics, while those with moderate (4–6) and severe (≥7) pain were managed by intravenous analgesics; starting with acetaminophen, followed by non-steroidal anti-inflammatory drugs, lastly, opioids. Wound infection was defined as the presence of wound discharge plus the isolation of a pathogenic microorganism in the fluid discharge.

### Sample size

This study was designed for superiority to compare two drainage methods in patients undergoing single-port VATS lobectomy. The required sample size of the current RCT was not calculated in the interest of the patients, and the trial was kept pilot with a relatively small sample size.

### Statistical analysis

Patient data were collected using Smart Health Tower’s database. The obtained data were analyzed via Statistical Package for the Social Sciences software 25.0. Qualitative data were presented as proportions and percentages and were compared using the Chi-square (X^2^) test or Fisher’s exact test (if two of the cells were less than five). Quantitative variables were analyzed by using an independent sample *t*-test, and the data were presented in the form of means and standard deviations. *P-*values of <0.05 were regarded as significant. Interim analysis was performed every 6 months to evaluate the outcome of both groups.

## Results

**Figure 1.**
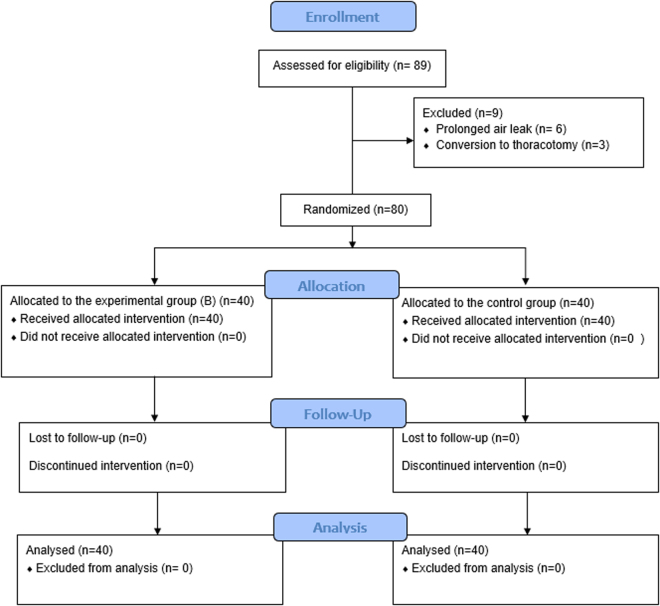
Flowchart of patient enrollment and inclusion.

The study included 89 patients. Six were excluded due to a prolonged air leak, and three were excluded due to intraoperative conversion to thoracotomy. Consequently, 80 participants (each group containing 40 patients) were included in the data analysis (Supplemental Digital Content, available at: http://links.lww.com/MS9/A795). Figure 1 illustrates the CONSORT flowchart for patient enrollment and inclusion. The sociodemographic, clinical, and surgical characteristics of the two arms were well-balanced. The mean age was 65.35 ± 9.47 years, 64 (80%) patients were male, 69 (86.25%) patients had 0 ECOG score, 11 (13.75%) patients had a score 1, 33 (41.25%) participants had a history of hypertension, and 57 (71.25%) patients had chronic obstructive airway disease (Table 1). The majority of the patients, 28 (35%) were in stage IIIA, 26 (32.5%) patients were in stage IIb, 10 (12.5%) patients were in stage IIa, 13 (16.25%) participants were in stage Ib, and 3 (3.75%) patients were in stage Ia. The mean duration of the operation was 181 minutes, and the average amount of blood loss was 241 mm (Table 2). There was no mortality in the 3 months; one patient from group A needed debridement and irrigation under general anesthesia and the insertion of a chest tube in a separate incision as the utility incision was discharging and sucking air. Concerning the outcome, there was a small but statistically significant difference in return to work when comparing group A with group B (34 ± 6 vs 31 ± 4 days, *P* = 0.038). More important were the differences in patients with wound discharge (29 vs 4; *P* < 0.001), differences in wound infection rate between both groups (16 vs 3; *P* = 0.001) and especially the need for intravenous antibiotic treatment (12 vs 1; *P* < 0.002, Table 3). There was only a minimal trend for a decrease in the duration of hospitalization. There was no significant difference in postoperative pain scores (Table 3). The comparison of pathogenic microorganism isolation between groups A and B revealed a significant difference (16 in group A compared to 3 in group B; *P*-value = 0.001) (Table 4).

**Table 1:** The Baseline characteristics of the participants

Characteristics	Group A	Group B	*P-value*
Mean age ± SD (year)	65.32 ± 9.43	65.37 ± 9.63	0.981
S, *n (%)*			0.264
Male	30 (75)	34 (85)	
Female	10 (25)	6 (15)	
ECOG performance status, *n (%)*			0.330
0	33 (82.5)	36 (90)	
1	7 (17.5)	4 (10)	
Smoking status, *n (%)*			0.288
Ever smoker	37 (92.5)	34 (85)	
Non-smoker	3 (7.5%)	6 (15)	
Pack years among ever smokers, *n (%)*			0.714
≥20	34 (91.9)	32 (94.1)	
<20	3 (8.1)	2 (5.9)	
Comorbidities, *n (%)*			
Hypertension	18 (45)	15 (37.5)	0.496
Chronic obstructive pulmonary disease	30 (75)	27 (67.5)	0.459
Cardiovascular system disease	11 (27.5)	7 (17.5)	0.284
Cerebrovascular accident	6 (15)	5 (12.5)	0.745
FEV1, %, mean ± SD	1741 ± 722	1882 ± 837	0.422
FVC, %, mean ± SD	2786 ± 866	2734 ± 860	0.788

*SD* standard deviation

**Table 2:** The surgical characteristics of the patients

Characteristics	Group A	Group B	*P-value*
Pathological type, *n (%)*			0.759
Squamous cell carcinoma	28 (70)	29 (72.5)	
Adenocarcinoma	11 (27.5)	9 (22.5)	
Other	1 (2.5)	2 (5)	
Cancer stage, *n (%)*			0.917
1a	1 (2.5)	2 (5)	
1b	7 (17.5)	6 (15)	
2a	6 (15)	4 (10)	
2b	13 (32.5)	13 (32.5)	
3a	13 (32.5)	15 (37.5)	
Tumor diameter, cm, mean ± SD	3.6 ± 1.2	3.7 ± 1.4	0.651
Operative duration, min, mean ± SD	179 ± 47	184 ± 49	0.633
Blood loss, ml, mean ± SD	250 ± 261	232 ± 268	0.722
Lobectomy type, *n (%)*			0.936
Right upper	13 (32.5)	13 (32.5)	
Left upper	8 (20)	8 (20)	
Right lower	9 (22.5)	11 (27.5)	
Left lower	10 (25)	8 (20)	
Lymph node dissection, *n (%)*			0.291
Systematic	27 (67.5)	21 (52.5)	
Sampling	7 (17.5)	10 (25)	
Hilar	6 (15)	9 (22.5)

**Table 3:** Postoperative outcomes of Groups A and B

Characteristics	Group A	Group B	*P-value*
Presence of discharge after chest tube removal, *n (%)*			<0.001
Yes	29 (72.5)	4 (10)	
No	11 (27.5)	36 (90)	
Required intravenous antibiotics, *n (%)*			0.002
Yes	12 (30)	1 (2.5)	
No	28 (70)	39 (97.5)	
Hospitalization duration, days, mean ± SD	4.6 ± 2.4	3. ± 0.9	0.079
Mean time of return to work ± SD (day)	34 ± 6	31 ± 4	0.038
Postoperative pain score, *n (%)*			0.490
Mild	7 (17.5%)	5 (12.5%)	
Moderate	21 (52.5%)	18 (45%)	
Severe	12 (30%)	17 (42.5%)	
Wound infection, n (%)			0.001
Yes	16 (40)	3 (7.5)	
No	24 (60)	37 (92.5)

**Table 4:** Microbial profile of the two groups

Characteristics	Group A	Group B	*P-value*
Presence of microorganism, *n (%)*			0.001
Yes	16 (40%)	3 (7.5%)	
No	24 (60%)	37 (92.5%)	
Type of microorganism, *n (%)*			
*Proteus vulgaris*	1 (6.3%)	0 (0%)	
*Enterobacter cloacae*	1 (6.3%)	0 (0%)	
*Staphylococcus epidermidis*	5 (31%)	1 (33.3%)	
*Staphylococcus aureus*	4 (24.9%)	2 (66.3%)	
*Polymicrobial infection*	2 (12.5%)	0 (0%)	
*Escherichia coli*	1 (6.3%)	0 (0%)	
*Bacillus circulans*	1 (6.3%)	0 (0%)	
*Alcaligenes faecalis*	1 (6.3%)	0 (0%)	

## Discussion

Currently, minimally invasive operations are being adopted in many operative procedures, and with thoracic operations, a VATS approach avoids creating a large wound, the use of a big retractor, and intercostal nerve injury^[^14,15^]^. The advantages and feasibility of this technique have been previously described in many investigations^[^16^]^.

Improving surgical outcomes relies not solely on better technology but also on better techniques. With the popularization of ERAS, more precise approaches are required peri-operatively to promote rapid recovery after operation^[^17^]^. Often, because of the debilitating effects of lung cancer, patients may reach the operating room in a compromised state. Such states, in addition to surgical trauma, may increase the risk of infection and lengthen the recovery period^[^18^]^.

Single-port VATS lobectomy for pulmonary cancers is typically performed through the mid- and anterior axillary lines in the fourth or fifth intercostal space^[^19,20^]^. After the operation, a drainage tube is placed to evacuate pleural fluid and/or air and to ensure expansion of the remaining lung to fill the intrathoracic space^[^21^]^. Although the management of chest tubes has advanced with surgical techniques, there is still much debate about the type, size, criteria for, and site of placement of chest tubes. These concerns are important because poor postoperative management can increase the duration of hospital stays as well as impact appropriate postoperative rehabilitation^[^22^]^.

Conventionally, two chest tubes are inserted in the basal and apical positions for pleural drainage. Recently, many investigations have shown a single tube to be more effective than two, but these results have not been obtained via single-port VATS^[^12^]^. When performing single-port VATS, a chest tube has usually been placed through the same incision as the VATS utility port (commonly through the fourth or fifth intercostal space). When using a single chest tube, in theory, placing it in a lower intercostal space (sixth or seventh) might be preferable, questioning whether it is sufficient to place a single chest tube through the utility incision because it may be less effective in pleural drainage, causing poor lung re-expansion and leading to a pneumothorax^[^23^]^. However, the literature lacks any objective evidence (retrospective or RCT) regarding the superiority of the placement of a single chest tube through a different incision. To the best of our knowledge, this is the first RCT to examine this aspect of chest tube management.

Several investigations have compared thoracic drainage methods with either one or two chest tubes or even without a tube^[^9,24–30^]^. In some studies, the patients have received conventional thoracotomies and no single-port VATS. Hence, management strategies for chest drainage after a single-port VATS need further exploration because minimal invasiveness not only decreases chest wall trauma but also decreases postoperative complications, especially during the rehabilitation period^[^10^]^.

Several studies have explored related aspects of drainage techniques and incision management in uniportal VATS. In a study by Palleschi *et al*^[^31^]^ an alternative technique for chest tube placement was introduced in uniportal VATS, aiming to enhance positioning and reduce complications. This approach involves creating a dedicated tunnel for insertion, emphasizing improved outcomes and postoperative aesthetics. Their findings underscore the significance of innovative strategies in postoperative care, emphasizing the need for optimized lung re-expansion and complication prevention in thoracic surgery discussions^[^31^]^.

The patients in group B returned to their normal lifestyle about 3 days earlier than in group A, probably explained by the presence of discharge and infection after chest tube removal in group A. The most important and convincing finding about the benefit of a separate site for chest tube insertion was the prevalence of wound infections in group A, which was five times greater in comparison to group B. In addition, despite the additional incision for chest tube insertion in group B patients, postoperative pain was similar between the two groups. This unexpected finding may have been due to the presence of discharge and infection in group A.

In this study, there was no mortality. This may be explained by the fact that risky patients (like) have been excluded from the study.

There are several severe limitations in this trial. First, it is a single-center, non-blinded trial. Second, a small sample size. Third, the volume of pleural fluid drainage was not measured. Fourth, we excluded diabetics, immunocompromised patients, and others with serious comorbidities who may behave differently in the postoperative period.

## Conclusion

The current pilot RCT showed that in patients undergoing a VATS lobectomy, single chest tube drainage has superior results when the tube is placed through a separate incision rather than through the utility incision because of a quicker postoperative recovery and fewer wound infections.

## Data Availability

The data that support the findings are available from the corresponding author, upon request.

## Research registration unique identifying number (UIN)

Research registry; the research was registered in the Research Registry. The registration number is researchregistry8426. The link is (https://www.researchregistry.com/registernow#home/?view_2_search=researchregistry8426&view_2_page=1).
